# Slaughterhouse Wastewater Treatment by Combined Chemical Coagulation and Electrocoagulation Process

**DOI:** 10.1371/journal.pone.0040108

**Published:** 2012-06-29

**Authors:** Edris Bazrafshan, Ferdos Kord Mostafapour, Mehdi Farzadkia, Kamal Aldin Ownagh, Amir Hossein Mahvi

**Affiliations:** 1 Health Promotion Research Center, Zahedan University of Medical Sciences, Zahedan, Iran; 2 School of Public Health, Tehran University of Medical Sciences, Tehran, Iran; 3 Center for Solid Waste Research, Institute for Environmental Research, Tehran University of Medical Sciences, Tehran, Iran; 4 National Institute of Health Research, Tehran University of Medical Sciences, Tehran, Iran; Queen's University Belfast, United Kingdom

## Abstract

Slaughterhouse wastewater contains various and high amounts of organic matter (e.g., proteins, blood, fat and lard). In order to produce an effluent suitable for stream discharge, chemical coagulation and electrocoagulation techniques have been particularly explored at the laboratory pilot scale for organic compounds removal from slaughterhouse effluent. The purpose of this work was to investigate the feasibility of treating cattle-slaughterhouse wastewater by combined chemical coagulation and electrocoagulation process to achieve the required standards. The influence of the operating variables such as coagulant dose, electrical potential and reaction time on the removal efficiencies of major pollutants was determined. The rate of removal of pollutants linearly increased with increasing doses of PACl and applied voltage. COD and BOD_5_ removal of more than 99% was obtained by adding 100 mg/L PACl and applied voltage 40 V. The experiments demonstrated the effectiveness of chemical and electrochemical techniques for the treatment of slaughterhouse wastewaters. Consequently, combined processes are inferred to be superior to electrocoagulation alone for the removal of both organic and inorganic compounds from cattle-slaughterhouse wastewater.

## Introduction

Wastewater from a cattle slaughterhouse is a mixture of the processing water from both the slaughtering line and the cleaning of the guts, which causes a large variation in the concentration of organic matter. The main pollutant in slaughterhouse effluents is organic matter. The contributors of organic load to these effluents are paunch, feces, fat and lard, grease, undigested food, blood, suspended material, urine, loose meat, soluble proteins, excrement, manure, grit and colloidal particles [1], [2].

Untreated slaughterhouses waste entering into a municipal sewage purification system may create severe problems, due to the very high biological oxygen demand (BOD) and chemical oxygen demand (COD) [3]. Therefore treating of slaughterhouse wastewater is very important for prevention of high organic loading to municipal wastewater treatment plants. The most common methods used for treating slaughterhouse wastewaters are fine screening, sedimentation, coagulation– flocculation, trickling filters and activated sludge processes.

The treatment of slaughterhouse wastewater by various methods such as aerobic and anaerobic biological systems [4], [5], [6], [7] and hybrid systems [2] have been intensively studied. Aerobic treatment processes are limited by their high energy consumption needed for aeration and high sludge production. Also, the anaerobic treatment of slaughterhouse wastewater is often slowed or impaired due to the accumulation of suspended solids and floating fats in the reactor which lead to a reduction in the methanogenic activity and biomass wash-out. In addition, it is also reported that anaerobic treatment is sensitive to high organic loading rates, as a serious disadvantage [8]. Even though biological processes are effective and economical, both biological processes require long hydraulic retention time and large reactor volumes, high biomass concentration and controlling of sludge loss, to avoid the wash-out of the sludge. Among physico-chemical processes, dissolved air flotation (DAF) and coagulation–flocculation units are widely used for the removal of total suspended solids (TSS), colloids, and fats from slaughterhouse wastewaters [1].

Chemical coagulation of slaughterhouse wastewater has also been studied by adding aluminum salts and polymer compounds, and a maximum COD removal efficiency of 45–75% has been reported [9], [10]. Polyaluminum chloride (PACl) is commonly used as the flocculant to coagulate small particles into larger flocs that can be efficiently removed in the subsequent separation process of sedimentation and/or filtration. Much attention has been paid to PACl in recent years because of its higher efficiency and relatively low costs compared with the traditional flocculants [11], [12]. On the other hand, PACl has become one of the most effective coagulant agents in water and wastewater treatment facilities with various applications, including removal of colloids and suspended particles, organic matter, metal ions, phosphates, toxic metals and color [13].

Recently, electrochemical methods such as electrooxidation [14] and electrocoagulation have been widely used as an attractive and suitable method for the treatment of various kinds of wastewater such as poultry and cattle slaughterhouse wastewater and wastewaters contain heavy metals, by virtue of various benefits including environmental compatibility, adaptability, energy efficiency, safety, selectivity, amenability to automation, and cost effectiveness [15], [16], [17], [18], [19], [20]. An examination of the chemical reactions occurring in the electrocoagulation process shows that the main reactions occurring at the electrodes (aluminum electrodes) are:
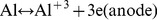
(1)

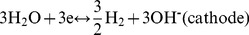
(2)


In addition, Al^3+^ and OH^−^ ions generated at electrode surfaces react in the bulk wastewater to form aluminum hydroxide:
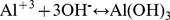
(3)


The aluminum hydroxide flocs normally have large surface areas which are beneficial for a rapid adsorption of soluble organic compounds and trapping of colloidal particles [15], [16], [21]. Also, these flocs polymerize further and are removed easily from aqueous medium by sedimentation or/and flotation by hydrogen gas.

Chemical coagulation using PACl and electrocoagulation process with aluminum electrodes of wastewater from a cattle slaughterhouse is described in this article. The purpose of this work was to investigate the feasibility of treating cattle-slaughterhouse wastewater by combined chemical coagulation and electrocoagulation process separately to achieve the required legal direct-discharge limit of COD and BOD_5_ which is 60 and 30 mg/L in Iran for the slaughterhouse industry effluents. The influence of the operating variables such as coagulant dose, pH, applied voltage and reaction time on the removal efficiencies of major pollutants was also determined. Information regarding the electrical energy consumption (EEC) is also included to provide an estimation of the cost of pollutants removal by an electrocoagulation system.

## Results and Discussion

### Wastewater characterization

Table 1 presents the slaughterhouse wastewater characteristics prior to any treatment, after 24 h settling time and the guidelines from Iran for effluent discharge in the sewage urban works. The values of the pollution parameters were lowered after 24 h of preliminary settling time. Also, the comparison of these values showed that, the COD, BOD_5,_ microbial indicators (Total and Fecal Coliforms) and the concentration of Oil and grease were very greater than those recommended by Iran. Consequently, the slaughterhouse effluent needed to be treated before discharge.

**Table 1:** Characteristics of the experimental cattle slaughterhouse wastewater.

Parameter	Raw wastewater Mean ± S.D.	24 h settled wastewater Mean ± S.D.	Permissive levels (Iran Standard for discharge to surface waters)
Number of samples	48	48	–
pH	7.31±0.12	7.44±0.16	6.5–8.5
Total COD (mg/L)	5817±473	4159±281	60
Total BOD (mg/L)	2543±362	2204±177	30
Total Suspended Solids (mg/L)	3247±845	1172±84	60
Total Kjeldhal Nitrogen (TKN) (mg/L)	137±12	92±12	–
Fat, oil, grease (mg/L)	34±9	32±7	10
Conductivity (μS/Cm)	9140±1512	9061±1400	–
Total Coliform (MPN/100 mL)	2.8×10^9^±1.5×10^7^	2.3×10^9^±2.6×10^7^	1000
Fecal Coliform (MPN/100 mL)	1.9×10^8^±2.1×10^6^	1.7×10^8^±2.3×10^6^	400

### Effect of preliminary settling time

Preliminary settling process is a natural treatment method that requires no chemical addition. Although some workers realized the importance of the natural settling process, there is little information available in the literature on the effect of the preliminary settling time on TSS removal capacity [22]. Most studies carried out on the treatment of slaughterhouse wastewater were based on diluted pre-settled wastewater [23].

In this study, the raw slaughterhouse wastewater was allowed to settle for 24 h in a preliminary settling tank before the addition of a coagulant. The process had an effect on BOD_5_, COD, TSS, TKN and coliform bacteria removals on the first 24 h. TKN concentration reduced from 137±12 to 92±12 mg/L (on average 33% TKN removal efficiency), COD concentration reduced from 5817±473 to 4159±281 mg/L (approximately 28% COD removal efficiency) whereas BOD_5_ was reduced in the wastewater from 2543±362 to 2204±177 mg/L (about 13% BOD_5_ removal efficiency). Furthermore, TSS concentration was reduced to 1172±84 mg/L (approximately 64% TSS removal efficiency). Similar results were reported by Amuda and Alade [10].

Also, data revealed that the effluent of the settling unit is characterized by high load of organic matter. The ratio BOD_5_/COD of approximately 0.5, indicates that 50% of the COD of this wastewater is easily able to be degraded by biological treatment. Nevertheless, the remainder COD is high, which indicates the necessity of an efficient physicochemical treatment for this wastewater.

### Effect of coagulation process (first step)

**Figure 1:**
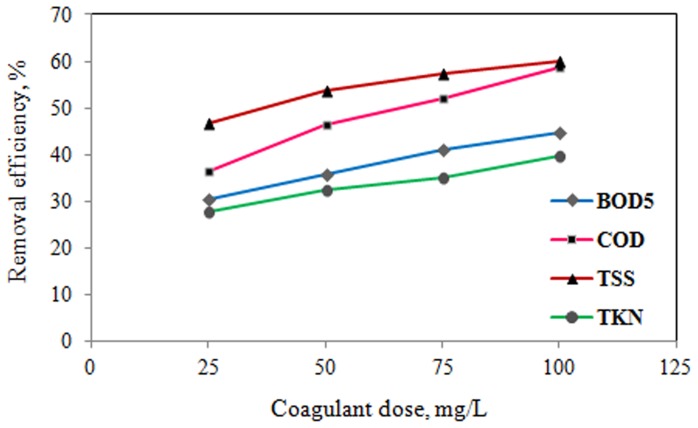
Effects of coagulant dose (PACl) on pollutants removal efficiency at pilot scale coagulation process.

Coagulation/flocculation experiments using PACl as coagulant in the jar test were performed to investigate the effect of coagulation process in the removal efficiencies of COD, BOD_5_, TSS, TKN and coliform bacteria. Therefore, PACl was added to the slaughterhouse wastewater to achieve particle instability and increase in the particle size, consequently achieving effective removal of organic substances present as COD and BOD_5_. The doses of PACl as coagulant were varied between 0 and 100 mg/L to determine the optimum dose of PACl for pollutants removal. The results of jar-tests using the PACl individually are presented in Table 2 and Figure 1. It is shown that at lower doses of the PACl (25 mg/L), COD, BOD_5,_ TSS and TKN removal efficiency reached a maximum of 37%, 31%, 47% and 27%, respectively. Aguilar et al. [24] reported TKN removal efficiency 50–60% by using PACl as coagulant from slaughterhouse wastewater. Also, Amuda and Alade [10] were reported maximum removal efficiency 65% and 34% of COD and TSS using a 750 mg/L dose of alum as coagulant in abattoir wastewater treatment.

**Table 2:** Influence of PACl dosage on water quality parameters of coagulated mixed liquor (mean values).

PACl dosage (mg/L)	Water quality parameters of treated effluent after chemical coagulation unit					
	COD (mg/L)	BOD_5_ (mg/L)	TSS (mg/L)	TKN (mg/L)	TC (MPN/100mL)	FC (MPN/100mL)
0	4159	2204	1172	192	2.3×10^9^	1.70×10^8^
25	2643	1534	623	139	2.8×10^7^	3.00×10^5^
50	2228	1418	544	130	1.6×10^7^	2.59×10^5^
75	2002	1301	501	125	2.3×10^6^	2.20×10^5^
100	1725	1217	470	116	1.6×10^6^	1.08×10^5^

As it shown in Figure 1, the efficiency of the process increased with increasing dosages of coagulant (PACl). The curve obtained with PACl points to a considerable increase in performance from the lowest dose up to 100 mg/L. On the other hand in chemical coagulation, as seen in Figure 1, an increase in COD, BOD_5_, TSS, TKN and other pollutants removal efficiency is noted with increasing PACl dosage, reaching nearly 40–60% for PACl dosage of 100 mg/L. Al-Mutairi et al. [9] reported that suspended solids and turbidity removal from slaughterhouse wastewater increased substantially as the alum (as coagulant) dosage is increased. Also, Figure 1 shows that the TSS removal and COD and BOD_5_ reduction trends are similar to each other. This may be due to the high organic contents of the suspended solid particles.

Maximum TC and FC removal efficiency of >99.9% (Table 2) were obtained by using PACl at the dosage of 100 mg/L. The TC and FC reduction, increase with increase in coagulant dosage.

TC indicator of effluent with coagulant dose 25 mg/L PACl was reduced from 2.3×10^9^ to 2.8×10^7^ (MPN/100mL) (approximately more than 98% TC removal efficiency), and by increasing the coagulant dose to 100 mg/L, the TC indicator of effluent was decreased was reduced from 1.7×10^8^ to 1.6×10^6^ (MPN/100mL) (on average more than 99% TC removal efficiency) that is much more than permissible level. A similar reduction trend was determined for FC indicator. Similar results were obtained in previous reports concerning the electrocoagulation of wastewater from vegetable oil refinery wastewater using aluminum electrodes (with adding PACl as coagulant aid) [19].

According the results of this study (Table 2) it can be concluded that although the efficiency for removal of most parameters from slaughterhouse wastewater are high, but the concentration of pollutants in effluent of chemical coagulation process does not meet the effluent discharge standards to the environment. Thus, the effluent from conventional coagulation should be preceded by another treatment process to be completed. For this purpose, in this research, electrocoagulation was employed as a completion of treatment process to obtain discharge standards.

### Effect of electrocoagulation process (second step)

Electrocoagulation processes a direct current source between metal electrodes immersed in wastewater. The electrical current causes the dissolution of metal electrodes commonly iron and aluminum into wastewater. The dissolved metal ions, at an appropriate pH, can form wide ranges of coagulated species and metal hydroxides that destabilize and aggregate the suspended particles or precipitate and adsorb dissolved contaminants [25], [26].

As be mentioned earlier, an examination of the chemical reactions occurring in the electrocoagulation process shows that the main reactions occurring at the aluminum electrodes are:
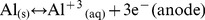
(4)

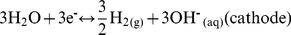
(5)

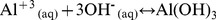
(6)


monomeric species such as Al(OH)^2+^, Al(OH)_2_^+^, Al_2_(OH)_2_^4+^, Al(OH)^4−^ and polymeric species such as Al_6_(OH)_15_^3+^, Al_7_(OH)_17_^4+^, Al_8_(OH)_20_^4+^, Al_13_O_4_(OH)_24_^7+^, Al_13_(OH)_34_^5+^ are formed during the electrocoagulation process [26], [27]. The aluminum hydroxide flocs act as adsorbents and/or traps for pollutants and so eliminate them from the solution [28], [29].

As mentioned earlier, the performances by the two pretreatment, namely, preliminary settling and chemical coagulation, were not carried out efficiently enough to satisfy the national guideline of effluent qualities. Additional dosage of coagulant (PACl) and longer time were needed to keep the national guideline of the effluent qualities. Therefore, the electrocoagulation process was employed as the final treatment step in this study. In adopting the electrocoagulation process, it was intended to treat the pollutant efficiently as well as economically.

**Figure 2:**
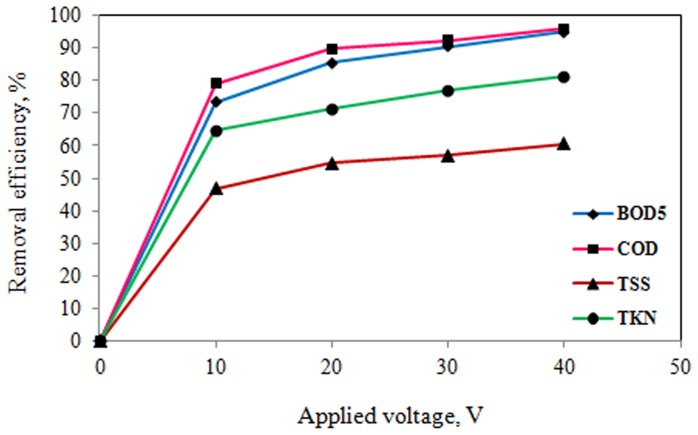
Effect of applied voltage on pollutants removal efficiency (coagulant dose: 25 mg/L, reaction time: 60 min).

**Figure 3:**
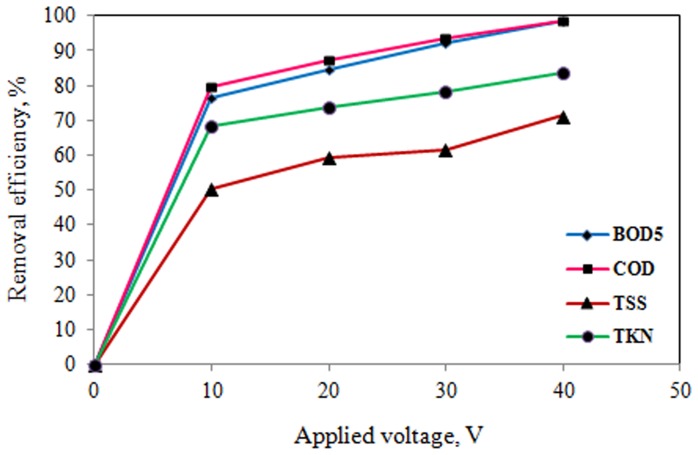
Effect of applied voltage on pollutants removal efficiency (coagulant dose: 50 mg/L, reaction time: 60 min).

**Figure 4:**
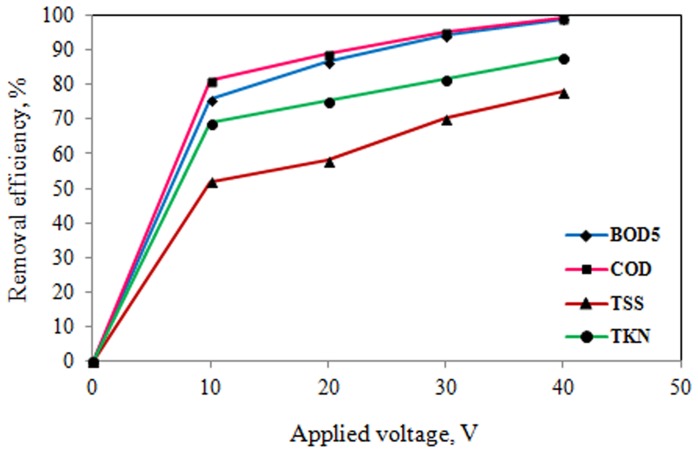
Effect of applied voltage on pollutants removal efficiency (coagulant dose: 75 mg/L, reaction time: 60 min).

**Figure 5:**
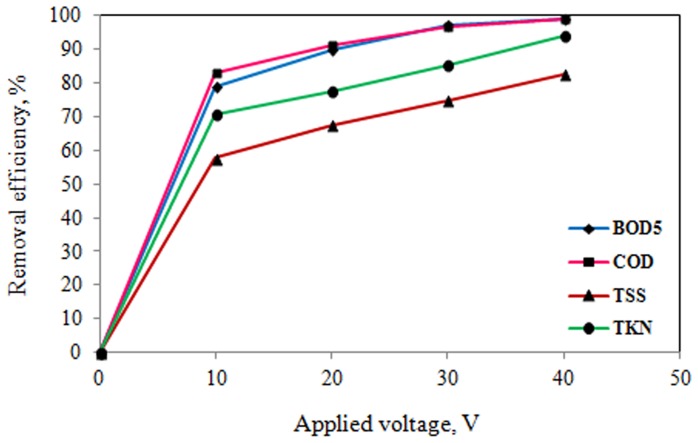
Effect of applied voltage on pollutants removal efficiency (coagulant dose: 100 mg/L, reaction time: 60 min).

The effects of applied voltage and reaction time on electrocoagulation process of slaughterhouse wastewater treatment were determined. The results of the effects of operating parameters on pilot scale electrocoagulation process are shown in Table 3 and Figures 2, 3, 4, and 5.

**Table 3:** Influence of electrocoagulation process using aluminum electrodes on effluent quality parameters (mean values).

PACl dosage (mg/L)	Applied voltage (V)	Water quality parameters of treated effluent after electrocoagulation unit					
		COD (mg/L)	BOD mg/L)	TSS (mg/L)	TKN (mg/L)	TC (MPN/100mL)	FC (MPN/100mL)
25	10	555	409	331	49	6743	1274
	20	267	223	282	40	6437	814
	30	203	146	268	32	4764	759
	40	108	79	245	26	4139	634
50	10	452	332	270	41	6274	1347
	20	283	218	221	34	5712	712
	30	145	108	209	28	4831	643
	40	29	21	156	21	4376	473
75	10	376	316	241	39	5712	785
	20	225	174	210	31	4833	531
	30	96	74	149	23	3157	375
	40	18	13	111	15	2563	114
100	10	294	254	199	34	3652	437
	20	153	125	153	26	2715	364
	30	59	33	118	17	1864	153
	40	13	10	82	7	943	72

### Effect of applied voltage

One of the most important parameter influencing the performance and economy of the electrocoagulation process is the applied voltage at the electrodes [30]. To understand the effect of applied voltage on the efficiency of electrocoagulation process in treating of slaughterhouse wastewater, several voltages in the range of 10 to 40 V were applied between the electrodes in the electrocoagulation cell, and pollutants removal was determined at the conditions given in Table 3.

The applied voltage is expected to exhibit a strong effect on electrocoagulation, especially on the COD abatement: higher the current (voltage), shorter the treatment. The supply of current to the electrocoagulation system determines the amount of Al^3+^ ion released from the respective electrodes and the amount of resulting coagulant. Thus, more Al^3+^ ion get dissolved into the solution and the formation rate of Al(OH)_3_ is increased. Also, it is well-known that electrical potential not only determines the coagulant dosage rate but also the bubble production rate and size and the flocs growth [31], [32], which can influence the treatment efficiency of the electrocoagulation process.

As it can be seen from Table 3 and Figures 2, 3, 4, and 5, the removal efficiency of pollutants is very high and as expected, it appears that for a given time, the removal efficiency increased significantly with increase of electrical potential. As the results shown in Table 3 and Figures 2, 3, 4, and 5, the removal efficiencies increased as the electrical potentials are increased. As an example, COD concentration of chemical coagulation process with 25 mg/L PACl has decreased from 2643 to 555 mg/L (approximately 79% COD removal efficiency) after electrocoagulation process with electrical potential of 10 V. Again, by increasing electrical potential to 40 V, the COD concentration in the effluent decreased to 108 mg/L in 60 min (approximately 96% COD removal efficiency). In addition, the COD of effluent from chemical coagulation with 100 mg/L PACl, was decreased to about 294 mg/L (approximately 83% COD removal efficiency) by electrocoagulation process with electrical potential of 10 V, and by increasing the electrical potential to 40 V, the COD of effluent was decreased to less than 13 mg/L (on average more than 99% COD removal efficiency) that is lower than permissible level.

According to the results of Table 3, and Figures 2, 3, 4, and 5, TKN of chemical coagulation process with 25 mg/L PACl was reduced to lower than 50 mg/L after electrocoagulation process with electrical potential of 10 V (approximately 65% TKN removal efficiency), and by increasing electrical potential to 40 V, the TKN concentration in the effluent decreased to 26 mg/L (about 81% TKN removal efficiency). Furthermore, with increase in coagulant dose to 100 mg/L and increase of applied voltage to 40 V, TKN concentration in effluent was reduced to lower than 7 mg/L (on average 94% TKN removal efficiency). A similar trend was seen for TSS and BOD_5_ parameters.

Also, as can be seen from Table 3, the removal efficiency of bacterial indicators (TC and FC) is very high and efficiency was increased with increase in applied voltage from 10 to 40 V. Maximum removal efficiency (>99.9%) was obtained in applied voltage 40 V (coagulant dosage 100 mg/L), and thus the effluent quality was reached to permissive levels (lower than 1000 and 400 for TC and FC, respectively) and hence discharge of this effluent to environment is safe. Also, minimum removal efficiency occurred in the lowest electrical potential (10 V). This is ascribed to the fact that at high voltage, the amount of aluminum oxidized increased, resulting in a greater amount of precipitate for the removal of pollutants. In addition, it was demonstrated that bubbles density increases and their size decreases with increasing current density [33], resulting in a greater upwards flux and a faster removal of pollutants and sludge flotation. As be mentioned earlier, the main mechanisms for removal of pollutants in this process are rapid adsorption of soluble organic compounds and trapping of colloidal particles in “sweep flocs” (Al(OH)_3_). Nevertheless, Bayar et al. [34] was reported that increase in the current density does not cause an expected removal efficiency increase; on the contrary, it can cause a relatively negative effect on it. Also, a similar trend was seen in the study of Holt et al. [32].

### Electrical energy and electrode consumption

**Figure 6:**
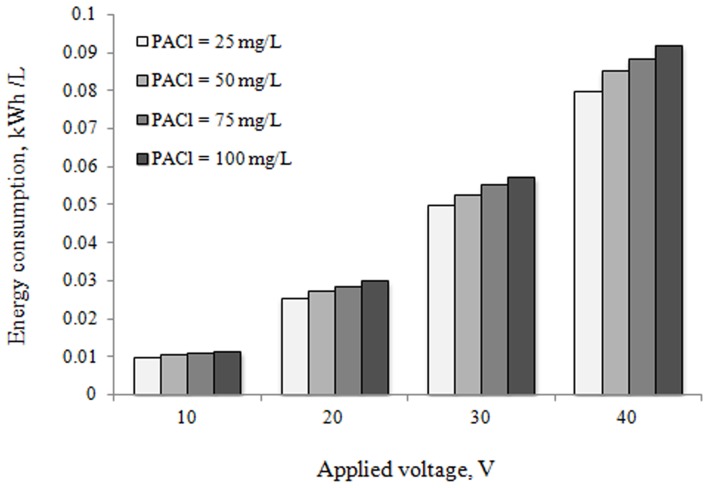
Electrical energy consumption during coagulation-electrocoagulation process (kWh/L).

**Figure 7:**
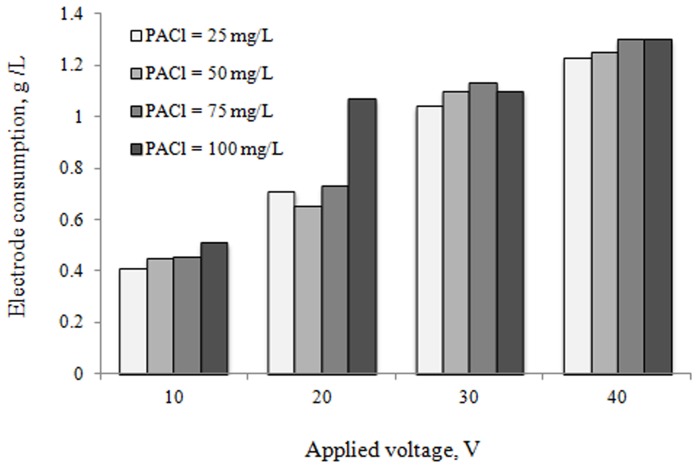
Electrode consumption during chemical coagulation-electrocoagulation process.

Electrical energy consumption is a very important economical parameter in the electrocoagulation process. Therefore, for the same operating conditions, after 60 min of electrocoagulation, consumption of energy and aluminum electrode is also represented in Figures 6 and 7. The electrical energy consumption was calculated using the related equations [35].

It can be understood from Figures 6 and 7 that electrical energy and electrode consumption were found to increase with increasing the applied voltage as would be expected in any other electrolytic process. An increase in applied voltage from 10 to 40 V causes an increase in energy consumption from about 0.001 to 0.08 kWh/L and from 0.011 to approximately 0.09 kWh/L for 25 and 100 mg/L of coagulant dosage (PACl), respectively. A similar trend was seen in the study of Bayar et al. [34] on Poultry slaughterhouse wastewater treatment by electrocoagulation method.

Also, as shown in Figure 7, an increase in applied voltage from 10 to 40 V causes an increase in electrode consumption from about 0.41 to 1.23 g/L and 0.51 to 1.3 g/L of pollutants for 25 and 100 mg/L of PACl, respectively. This result is in agreement with the results obtained by Bazrafshan et al. [36], [37], [38].

When the applied voltage was increased from 10 V to 40 V, the COD and BOD_5_ removal efficiency increased appreciably, to more than approximately 99%, whereas the corresponding specific energy consumption increased only slightly. Therefore, in present study, 40 V is chosen as optimum operating voltage for electrocoagulation process.

### Conclusions

In this study, chemical coagulation using Polyaluminum chloride (PACl) and Electrocoagulation process using aluminum electrodes of wastewater from a cattle slaughterhouse was investigated. The effects of the different operational parameters on the removal of pollutants analyzed. The following conclusions can be reached from the results obtained in this work:

The installation of a good fat separator prior to each biological or chemical treatment unit seemed an appropriate alternative to a chemical coagulation and electrocoagulation process.
Preliminary settling time were investigated and found to be important operational parameter for effective treatment of slaughterhouse wastewater.
A preliminary settling time of 24 h had an effect on the BOD_5_, COD, TSS and TKN with removal efficiency up to 14%, 29%, 64% and 33%, respectively.
According to the results obtained from the present experiments, the removal efficiencies increased by increasing the coagulant dose and electrical potential. At the highest applied voltage, the fastest treatment rate for pollutants (COD, BOD_5_, TSS, TKN and microbial parameters) removal was obtained. Moreover, the energy consumption increased by increasing the applied electrical potential.
Evaluation of the experimental results indicates that both processes (chemical coagulation and Electrocoagulation) show excellent efficiency at reducing of pollutants.
Based on this study results, although the coagulation process had high efficiency in removing organic and microbial contaminants, but nevertheless it’s not able to meet discharge standards, hence a supplemental process (such as electrocoagulation process) is essential for enhance effluent quality.
Finally according to the results of this study, it can be concluded that the combined application of chemical coagulation and electrocoagulation processes is able to meet effluent standards for safe discharge to environment.

## Materials and Methods

### Slaughterhouse effluent

The effluent used throughout this study was taken from a local cattle Slaughterhouse plant with 250 cows per day capacity, located in Zahedan City in the province of Sistan and Baluchestan province (Iran), producing approximately 60 m^3^ of wastewater daily. The cattle slaughterhouse effluent was sampled after the screening of coarser solids using a filter having a pore size of approximately 2.0 mm and sedimentation for 24 h. Samples were collected in polypropylene bottles, shipped cold, and kept at 4°C before use. The length of the storage before starting experiments varied from one day to six weeks. The effluent has been sampled at different times during this study and the initial characteristics varied with time (Table 1). This effluent initially contained high concentrations of soluble and undissolved organics (4159±81 mg/L COD, 2204±77 mg/L BOD_5_).

### Chemical treatment (coagulation) of slaughterhouse effluent

All the chemicals used in the study were of analytical reagent (AR) grade. Poly- aluminum chloride (PACl) Al_12_Cl_12_(OH)_24_ was chosen for this study because it has been used extensively at water and wastewater treatment plants to remove solids and may function as an effective and less expensive coagulant. PACl was used in this study up to 100 mg/L (25, 50, 75 and 100 mg/L). A six-beaker jar test (flocculator) was set up at room temperature for each trial. Each of the beakers contained 2 L of settled wastewater. The coagulants were added into the beakers, and the pH values were immediately adjusted to the preset values (7±0.1) using NaOH or H_2_SO_4_ for pH-controlled experiments. Rapid stirring at 150 rpm for 2 min was followed by gentle mixing at 50 rpm for 20 min, and the solids formed were left to settle for 30 min. Samples were taken from the water surface (supernatant) and filtered through 0.45-mm membranes. After chemical coagulation, electrocoagulation process with aluminum electrodes was performed on the supernatant.

### Electrochemical treatment of slaughterhouse effluent

**Figure 8:**
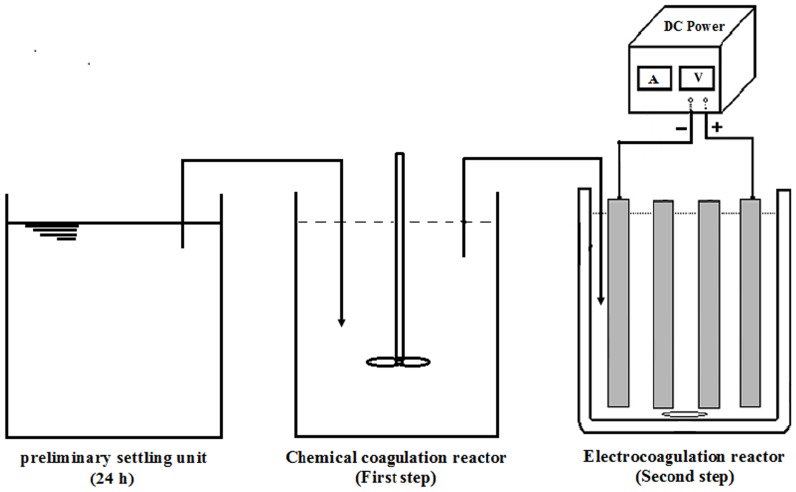
The schematic view of coagulation and electrocoagulation reactor.

In each run, wastewater (supernatant) after chemical coagulation (first stage of treatment) was poured into the electrocoagulation cell. All experiments were performed in a bipolar batch reactor (Figure 8), with four aluminum electrode connected in parallel. Only the outer electrodes were connected to the power source, and anodic and cathodic reactions occurred on each surface of the inner electrode when the current passed through the electrodes. The internal size of the cell was 15 Cm×15 Cm×25 Cm (width × length × depth) with an effective volume of 2000 Cm^3^. The volume (V) of the solution of each batch was 2 L. The active area of each electrode (plate) was 14×20 Cm with a total area of 280 Cm^2^. The distance between electrodes was 1.5 Cm. A power supply having an input of 220 V and variable output of 0–40 V (10, 20, 30 and 40 V) with maximum current of 5 ampere was used as direct current source. The temperature of each system was maintained at 25±1°C. Different samples of 100 ml were taken at 15 min intervals for up to 1 h and filtered before being analysed to determine BOD_5_, COD, TSS and other parameters. During the runs, the reactor unit was stirred at 150 rpm by a magnetic stirrer to allow the chemical precipitate to grow large enough for removal. During electrocoagulation, an oxide film formed at the anode. In order to overcome electrode passivation at the anode, the electrodes were rinsed in diluted HCl solution (5% v/v) after each experiment and rinsed again with tap water and finally weighted. Also the electrodes reweighted to calculate sacrificial electrode consumptions. These weights are used in the calculations of the total operating cost. In addition, the electrical energy consumed per unit volume of treated wastewater has been calculated for different experimental conditions. All analyses were conducted in duplicate for reproducibility of the experimental results, and all of the data in the Figures and Tables were the average ones.

### Analytical

COD, BOD_5_, oil-grease, conductivity, pH, total solids (TS), total suspended solids (TSS), and total Kjeldhal nitrogen (TKN) determinations were determined according to the standard methods [39]. COD was measured using COD reactor and direct reading spectrophotometer (DR/5000, HACH, USA). Five-day biological oxygen demand (BOD_5_) was determined by the manometric method with a respirometer (BSB-Controller Model 620 T (WTW)). Oil-grease was determined with hexane extraction. The pH and conductivity were adjusted to a desirable value using NaOH or H_2_SO_4_, and NaCl, and measured using a pH meter model E520 (Metrohm Herisau, Switzerland) and a Conductivity Meter (Jenway Model 4200), respectively. Also the most-probable-number technique was used for the enumeration of total coliform (TC) and fecal coliform (FC) bacteria [39].
